# Complex Presentation of Uterus Didelphys With Bilateral Leiomyomas: A Case Report

**DOI:** 10.1155/crog/6231298

**Published:** 2025-01-21

**Authors:** Assaye Mezgebu Wube, Yusuf Mohammed Yusuf, Biniyam Afework Abate, Meksud Shemsu Hussen, Yabets Tesfaye Kebede, Bekri Delil Mohammed

**Affiliations:** ^1^Department of Medicine, Ethio Tebib Hospital, Addis Ababa, Ethiopia; ^2^Department of Medicine, Jimma University, Jimma, Ethiopia

## Abstract

Müllerian duct anomalies and uterine leiomyomas represent distinctive facets of female reproductive health. While uterine leiomyomas are prevalent reproductive pathologies, the coexistence of Müllerian anomalies and leiomyomas is relatively uncommon. This case study examines the complex medical and surgical management of a woman who initially presented with chronic abdominal pain and swelling. Following evaluation, the patient received a diagnosis of an uncommon co-occurrence of congenital uterus didelphys and leiomyomas. Notably, during the surgical procedure, a rectovesical peritoneal band was identified. This case study seeks to broaden the scientific understanding of these subsets of women, offering insights into the complexities arising from a common diagnosis overlaying a rare condition.

## 1. Introduction

Müllerian duct anomalies are rare female reproductive tract developmental anomalies [1]. In our case, the identified Müllerian anomaly is uterus didelphys, characterized by the complete duplication of uterine horns and cervix with the presence of a longitudinal vaginal septum [2]. It results from an incomplete or complete arrest in the midline fusion of Müllerian ducts [3], whereas uterine leiomyomas are distinct pathologies characterized as benign monoclonal tumors that originate from the smooth muscle cells and fibroblasts of the myometrium [4].

The incidence of Müllerian anomalies is estimated to range from 0.1% to 10% [5]. Uterus didelphys accounts for approximately 8%–11% of Müllerian anomalies [6]. Conversely, leiomyomas affect up to 70%–80% of females [7]. With the provided figures, the probability of both occurrences happening simultaneously becomes rare [8].

Patients with Müllerian anomalies typically remain asymptomatic before puberty, with most symptoms emerging during childbearing age [9]. Common manifestations include infertility, dyspareunia (especially when a vaginal septum is present), periodic lower abdominal pain (attributed to the retention of blood in the noncommunicating uterine segment), and menorrhagia (resulting from an increased surface area of the endometrium) [3, 10]. Additionally, these patients face risks of obstetric complications such as cervical incompetence, ectopic pregnancy, preterm labor, and malpresentation [11, 12]. Müllerian anomalies often coexist with congenital anomalies, particularly in the urologic domain (e.g., renal agenesis, pelvic kidney, and horseshoe kidney) and skeletal anomalies (e.g., cervicothoracic somite dysplasia) [13, 14]. Notably, uterus didelphys is more commonly associated with renal agenesis than other subtypes [3]. Herlyn–Werner–Wunderlich syndrome, which manifests as a triad of uterus didelphys, obstructed hemivagina, and ipsilateral renal agenesis, is another example [15].

Moreover, this case affords nuanced perspectives on the intricacies that may emerge from the simultaneous presence of these two conditions, elucidating the challenges encountered in radiologic imaging when both congenital and acquired factors converge. Noteworthy is the course of the sigmoid colon, wherein it traverses between the two uterine horns and attaches to the apex of the bladder. This comprehensive account seeks to contribute substantively to the scientific discourse and may serve as a reference point for medical practitioners navigating similar complex clinical scenarios.

## 2. Case History

A 32-year-old nulliparous married woman presented with a chief complaint of chronic dull lower abdominal pain persisting for 7 years, accompanied by a gradual onset of abdominal distension over a 5-year duration. Notably, she reported cyclic, heavy menstrual bleeding with clot formation lasting a week, devoid of associated pain during menstruation.

A previous history reveals no prior successful or failed pregnancies, despite a previous attempt at conception. She had no history of constitutional symptoms such as anorexia, weight loss, or a history of abdominopelvic surgery. The patient denied any medication use or prior contraceptive methods. She sought medical attention at another facility, where she was told to have an ovarian tumor with a recommendation for surgical intervention. However, she declined the proposed operation and sought treatment at our institution.

Upon physical examination, the patient's vital signs were within normal limits. Abdominal and genitourinary examinations disclosed an abdominopelvic mass equivalent to the size of a 28-week gravid uterus. The mass exhibited characteristics of firmness, nodularity, and limited mobility. Pelvic examination revealed no observable swellings, ulcers, or abnormal discharges. A digital vaginal and speculum examination revealed a double vaginal canal with a complete longitudinal septum between the two, wherein the right side exhibited greater width than the left. Both canals had distinct cervices, which were soft and nontender. No abnormal discharges or blood were detected during the digital examination. Other systemic examinations failed to yield any additional clinically significant findings.

The patient underwent a comprehensive diagnostic workup, including CBC, comprehensive metabolic panel, urine analysis (U/A), beta-human chorionic gonadotropin (B-HCG), venereal disease research laboratory (VDRL), provider-initiated counseling and testing (PICT), hepatitis B surface antigen (HBsAg), and cancer antigen 125 (CA 125), all yielding results within normal ranges. Abdominopelvic ultrasound revealed an enlarged uterus with multiple well-defined hypoechogenic intramural and subserosal masses, the largest of which was 10 × 9 cm. The endometrium, although only partially visualized, appeared normal. No masses were observed in the adnexa, and the Douglas pouch showed no fluid or mass. Normal-sized kidneys with normal parenchyma and sinus echo patterns, with no focal lesions or calculi detected. Mild calyceal dilation was noted in the right kidney.

**Figure 1:**
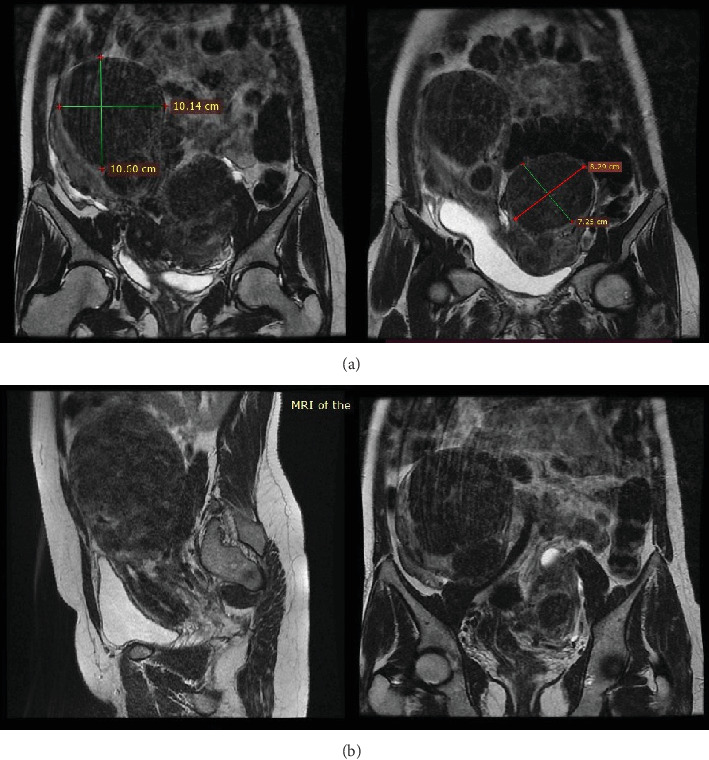
(a, b) MRI showing the initial finding of 10.14 × 10.6 cm mass in the right horn and 7.25 × 8.29 cm in the left horn. (b) Two distinct cervices with an upper vaginal septum.

In light of the contradiction between the initial diagnostic findings obtained at another medical facility and the pelvic examination revealing a double vagina with a longitudinal septum, suspicion arose regarding a potential Müllerian anomaly. To further elucidate the anatomical intricacies and gain a comprehensive understanding, an abdominopelvic MRI was subsequently conducted. The MRI revealed two distinct and widely separated uterine bodies and cervices, with an upper vaginal septum (see Figure 1). Both cervices exhibited normal outline and signal intensity. The endometrium displayed a normal signal intensity with preserved zonal anatomy. Multiple masses, at least five in each uterine horn, were identified as heterogeneously enhancing on T1 and hypointense on T2 imaging. The masses varied in size, with the largest in the right horn measuring 10.14 × 10.6 cm and the largest in the left horn measuring 7.25 × 8.29 cm. No adnexal masses were observed, and the posterior cul-de-sac was free of abnormalities. Both kidneys were visualized in a normal position without evidence of urinary drainage obstruction.

The patient's diagnosis was thoroughly discussed, emphasizing the presence of multiple uterine masses and the unique anatomical anomaly of having two uteruses. Given the patient's expressed desire to preserve fertility, potential management plans were obtained, and a decision was made to proceed with a myomectomy.

**Figure 2:**
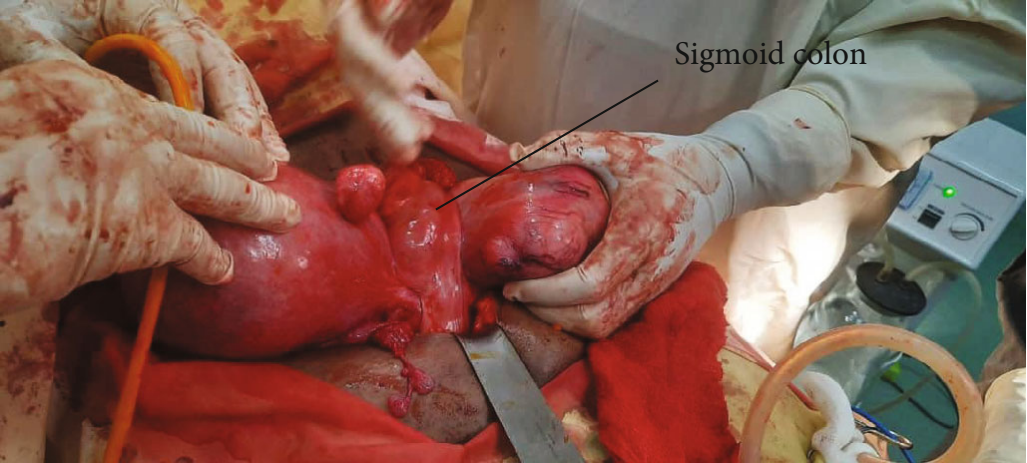
Visual representation depicting the sigmoid colon traversing between the two uteruses and attaching to the bladder apex.

**Figure 3:**
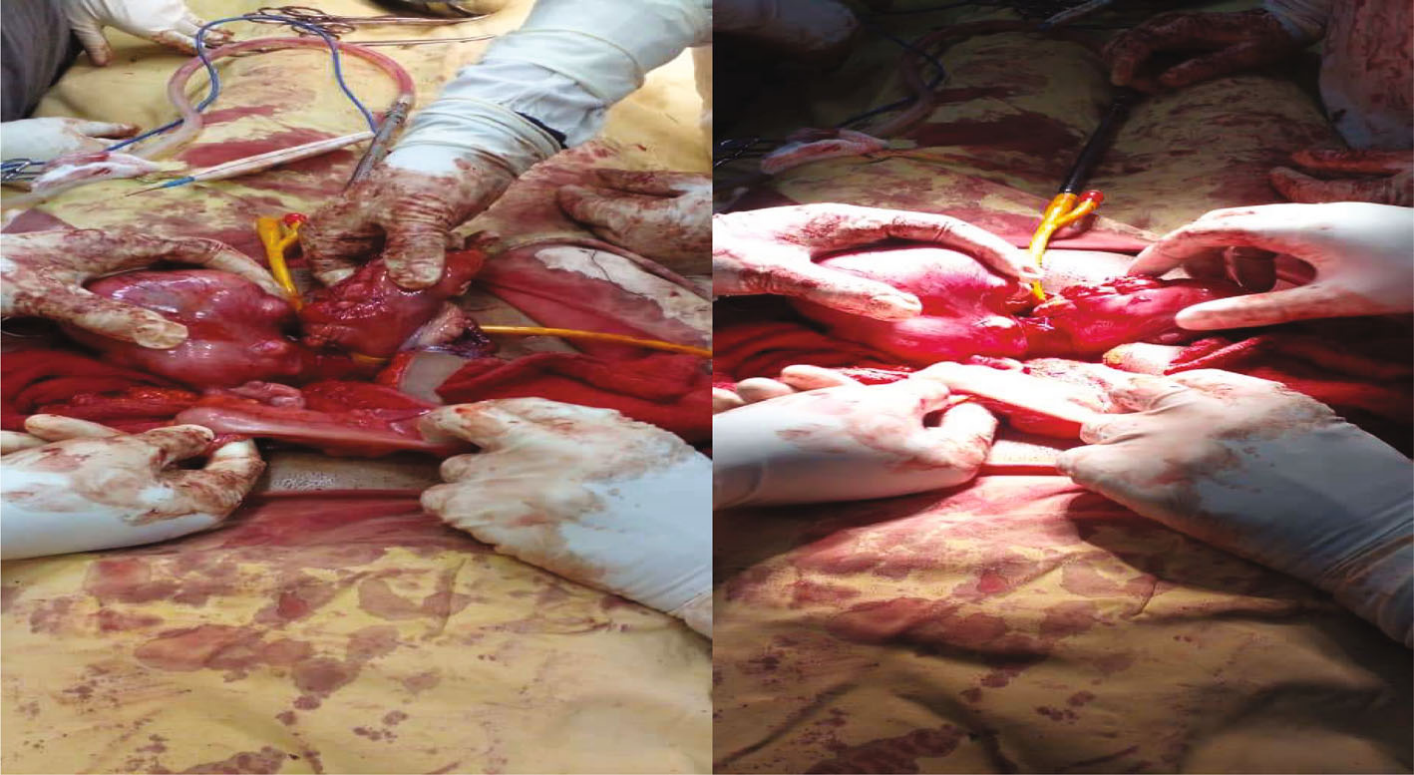
Image illustrating the postprocedure status following sigmoid colon release and myomectomy underway.

During the procedure, the patient was positioned supine, and the abdomen was meticulously prepared with an alcohol and iodine solution and then draped. An infraumbilical midline incision was made, entering the abdomen in layers. Intraoperatively, the finding unveiled a double uterus, each hosting multiple intramural and subserosal myomas of different sizes. Both ovaries and bilateral fallopian tubes appeared anatomically normal and appropriately positioned. Notably, there were two distinct cervices, and the sigmoid colon was found traversing in between the two uteruses, attaching to the isthmus of both uteruses and the apex of the bladder anteriorly by its mesentery (see Figure 2). In response to this finding, a general surgeon was promptly consulted and joined the operation. The sigmoid colon was carefully released, and a Foley catheter tourniquet was separately applied to each uterus. Three intramural myomas were individually excised from each uterus; the biggest sizes were 10 by 9 cm on the right uterus and about 8 by 7 cm on the left uterus; the rest were smaller and subserosal (see Figure 3). Biopsy samples were meticulously collected and forwarded to pathology for analysis. Meticulous hemostasis was achieved, obliterating potential dead spaces. An intraoperative dye test was performed, revealing spillage into the peritoneal cavity, peritubal, and adnexal structures. Before the layered closure of the abdomen, an instrument and gauze pack count were confirmed by the surgical team. The estimated blood loss after the procedure was 300 mL.

Postoperatively, the patient was placed on nothing by mouth (NPO) and received maintenance fluids. Antibiotic prophylaxis was already initiated, and analgesics were administered as needed. Vital signs were closely monitored, and no postoperative complications were observed during the recovery period.

**Figure 4:**
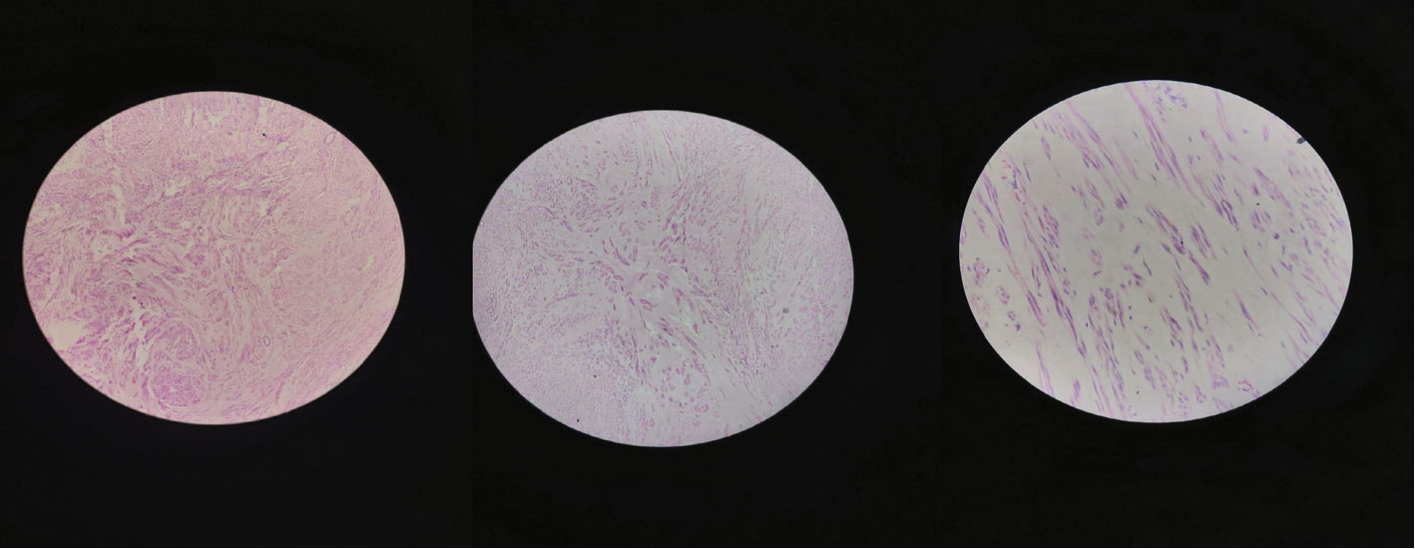
Histopathological image shows the proliferation of smooth muscle cells with uniform nuclei in a hyalinized stroma and infrequent mitotic activity, confirming the diagnosis of leiomyoma.

Upon receiving the biopsy results, it was confirmed that the pathology exhibited a proliferation of smooth muscle cells with uniform nuclei in a hyalinized stroma (see Figure 4). Mitotic activity was infrequent, leading to the conclusion of uterine leiomyoma. Subsequently, the patient was discharged on the third postoperative day, with a follow-up appointment scheduled after a week. The discharge medications included ferric ammonium citrate with folic acid syrup and analgesics as needed.

During the follow-up visit, the patient presented in a well-looking and good postoperative condition. She reported intermittent mild pain at the wound site as her sole complaint. Vital signs remained within the normal range, and the wound site was clean. The patient was provided counseling regarding future fertility options, with a plan for in vitro fertilization (IVF) in the event of unsuccessful attempts at natural conception and planned delivery via cesarean section. Follow-up appointments were scheduled to monitor and further discuss the patient's reproductive plans.

## 3. Discussion

The simultaneous presence of uterus didelphys and myoma has been reported, although infrequently [8, 16]. Our comprehensive literature review reveals a solitary African contribution to this subject [17]. This paucity of available reports may be attributed to the rare nature of such co-occurrences [8].

Diagnosing the concurrent presence of uterus didelphys and leiomyoma poses a considerable challenge. In our case, the abnormalities were not easily discernible on ultrasound, so the diagnosis was resolved with an MRI. The intricacy arose from multiple myomatous masses altering the uterine outline, rendering ultrasound less effective in obtaining a definitive diagnosis. Ali et al. [16] similarly encountered diagnostic complexity, with uterus didelphys being definitively diagnosed intraoperatively. This underscores the necessity for heightened vigilance and additional imaging modalities when suspicions of Müllerian anomalies are raised during assessments, particularly following suggestive pelvic examinations.

While ultrasound remains a commonly utilized diagnostic tool owing to its simplicity, noninvasiveness, and cost-effectiveness, its diagnostic efficacy is heavily contingent upon the expertise of the examiner [18]. The integration of three-dimensional (3D) ultrasound has demonstrated enhanced reliability, offering comprehensive evaluations of the cervix and vagina compared to conventional two-dimensional (2D) ultrasound methods [19, 20]. Despite its expense, MRI is considered the gold standard, providing precise 3D images detailing genital and peritoneal anatomy [21–23]. Moreover, MRI facilitates the assessment of potential associated congenital anomalies, particularly those of urological origin. Alternative imaging modalities include hysterosalpingo-contrast-sonography, x-ray hysterosalpingography, hysteroscopy, and laparoscopy [18, 24, 25]. However, their utility is limited by the associated drawbacks. The choice of imaging modality should be judiciously made, considering the specific clinical context and the need for comprehensive diagnostic accuracy.

Notably, despite employing MRI as an advanced imaging modality, the identification of the course of the sigmoid colon eluded detection and was only fortuitously discovered intraoperatively [26]. This highlights the imperativeness of interdisciplinary collaboration, prompting consultation with general surgery. After the collaborative effort, the sigmoid colon was released, bringing attention to the need for vigilance and meticulous exploration during surgery to uncover potential anomalies that might not be apparent on preoperative imaging. This unforeseen discovery underscores the importance of approaching each case with an open-minded perspective, anticipating the unexpected, and adapting surgical plans accordingly.

Our proposed course of action for this patient entails initially awaiting natural conception, with the expectation that the myomectomy procedure has addressed any underlying infertility issues. Subsequently, if natural conception proves unsuccessful, we plan to proceed with IVF, followed by a cesarean delivery. Noteworthy achievements in the realm of assisted reproductive technologies for individuals with a uterus didelphys have been documented [27]. The prospect of a twin pregnancy, with each uterus accommodating one fetus, is within the realm of consideration [28]. However, it is crucial to acknowledge the heightened susceptibility to obstetric complications associated with pregnancies in individuals with Müllerian anomalies, encompassing preterm delivery, premature rupture of the membranes, placental anomalies, malpresentation, and various obstetric challenges. Consequently, the potential complexities inherent in vaginal delivery in the context of Müllerian anomalies underscore the importance of a cautious approach and consideration of alternative delivery methods [27, 29].

## 4. Conclusions

This case report sheds light on the remarkable coexistence of uterus didelphys and leiomyomas, presenting a rare and intricate clinical scenario. Our findings underscore the importance of heightened vigilance, interdisciplinary collaboration, and meticulous exploration during surgery to reveal unexpected anomalies. As we navigate the complexities of diagnosing and managing such rare conditions, this study contributes valuable insights to the scientific discourse, serving as a reference for healthcare professionals confronted with similar challenges in reproductive health.

## Data Availability

All the data underlying the case report is available as part of the article.

## Nomenclature

CBC: complete blood count
U/A: urine analysis
B-HCG: beta-human chorionic gonadotropin
VDRL: venereal disease research laboratory
PICT: provider-initiated counseling and testing
HBsAg: hepatitis B surface antigen
CA 125: cancer antigen 125
NPO: nothing by mouth
DNS: dextrose and saline
IV: intravenous
IVF: in vitro fertilization
MRI: magnetic resonance imaging
2D: 2-dimensional
3D: 3-dimensional
